# NETTAB 2013: Semantic, social, and mobile applications for bioinformatics and biomedical laboratories

**DOI:** 10.1186/1471-2105-15-S14-S1

**Published:** 2014-11-27

**Authors:** Paolo Romano, Nicola Cannata

**Affiliations:** 1 Bioinformatics, IRCCS AOU San Martino - IST National Cancer Research Institute, Genoa, I-16132, Italy; 2 School of Science and Technology, University of Camerino, Camerino (MC), I- 62032, Italy

## Abstract

The thirteenth NETTAB workshop, NETTAB 2013, was devoted to semantic, social, and mobile applications for bioinformatics and biomedical laboratories.

Topics included issues, methods, algorithms, and technologies for the design and development of tools and platforms able to provide semantic, social, and mobile applications supporting bioinformatics and the activities carried out in a biomedical laboratory.

About 30 scientific contributions were presentedat NETTAB 2013, including keynote and tutorial talks, oral communications, and posters. Best contributions presented at the workshop were later submitted to a special Call for this Supplement.

Here, we provide an overview of the workshop and introduce manuscripts that have been accepted for publication in this Supplement.

## NETTAB workshops

Network Tools and Applications in Biology (NETTAB) Workshops are a series of International meetings held annually in Italy [1] whose goal is the analysis of the impact that some of the most innovative Information and Communication Technologies (ICTs) may have on biomedical research, and in particular on bioinformatics. Usually, scientific sessions are focused on tools, systems, applications and perspectives of their adoption. Discussion is a key factor within sessions and in special Panel Discussion sessions. A poster session allows all participants to present and discuss their projects and ideas on the main topics. The agenda of NETTAB workshops is completed by tutorials.

The workshop topics evolve, trying to cope with technology innovation. Each year the meeting is devoted to a different ICT technology or application domain. As a consequence, many different topics have been faced and discussed since 2001, some of which have been very timely, including XML for data integration (Genoa, 2001), multi-agent systems (2002, Bologna), scientific workflows (Naples, 2005), GRID and Web Services (Santa Margherita di Pula, 2006) [2], Semantic Web (Pisa, 2007) [3], collaborative and social tools for research (Catania, 2009) [4], biological Wiki systems (Naples, 2010) and on-line integrated bio-search (Como, 2012) [5].

## NETTAB 2013: the thirteenth edition

NETTAB 2013, thirteenth workshop in the series, was held in Lido of Venice, Italy, on October 16-18, 2013. It was organized by Nicola Cannata, University of Camerino, and Paolo Romano, Cancer Comprehensive Center and University Hospital San Martino IST, Genova, with support from Barend Mons, Leiden University Medical Center and Netherlands Bioinformatics Center, The Netherlands, and Andrea Splendiani, intelliLeaf, United Kingdom.

The idea behind the topic of workshop stands in the consideration that in last years ICTs have permeated human society of new ways of participation in social activities. In the Internet, the hype has shifted from Web2.0 to Social Media, which facilitate communication and make both data exchange and information and knowledge sharing easier. In biology, and especially in the "-omic" disciplines, we already rely on a wide diffusion of social tools and applications, e.g for distributed annotations, Wiki knowledge bases, documentation and productivity.

On the other hand, access to the Internet is nowadays increasingly happening through mobile devices. Although exact figures do not still exist, mobile Internet access is expected to soon overtake access from standard personal computers and workstations, while mobile phones are expected to become the main personal computing device soon. Smartphones and tablets seem to represent the most practical computing device in biomedical laboratories and to be the ideal companions for "always on the move" scientists. While we can observe a widespread diffusion of mobile applications related to health and lifestyle, as well as a rapid adoption of mobile solutions in medicine and healthcare, we cannot say the same for life sciences and bioinformatics. On the contratry, semantic methodologies and technologies are well established in "-omic" projects. It can even be proudly observed that the bioinformatics community was an early adopter of Semantic Web technologies.

The Scientific programme of the workshop included keynote lectures, well representing the main focus themes, and 12 oral communications; 8 posters were also presented at the workshop. The Proceedings were published by EMBnet.journal [6].

Three keynote talks were given. Antony Williams, of the Royal Society of Chemistry, gave a talk on "Facilitating Scientific Discovery through Crowdsourcing and Distributed Participation", where he pointed out the possible roles of collaboration among chemists, and researchers, in data curation [7]. He showed how data quality enhancements can come through crowdsourcing and intelligent robots. In this context, participation is essential and may be driven by new approaches to rewards and recognition.

Ross D. King, from the University of Manchester, gave its contribution on "Semantic technologies for the automation of research in biomedicine". His lecture introduced first the concept of a Robot Scientist, a physically implemented robotic system that applies techniques from artificial intelligence to execute cycles of automated scientific experimentation [8], and then the Robot Scientists Adam (for functional genomics), and Eve (for drug design). His vision of the future sees a collaboration between researchers (human scientists) and Robot Scientists able to improve science. In this context, the scientific knowledge will be expressed in logic with associated probabilities and published using the Semantic Web.

Finally, "SCIMOBS: the million minds approach revisited in mobile context" was the title of the lecture given by Barend Mons, Leiden University Medical Center and Netherlands Bioinformatics Center. In his talk, he revisited the 'need to engage a million minds in expert crowd sourcing', based on his 2008 paper [9], in the context of the nano-publication concept. In his opinion, now that mobile technology is so advanced both in technical development and in social acceptance, and the first real scientific applications are reaching the market, there is a need to revisit how to engage people in expert crowdsourcing.

Tutorials were given by Andrea Splendiani, IntelliLeaf, United Kingdom, who introduced vision, tools, and platforms for a Semantic Web for Life Sciences, Christine Chichester, Swiss Institute of Bioinformatics, who presented the Open PHACTS project and NanoPublications technologies and tools, Dominique Hazaël-Massieux, W3C/ERCIM, who introduced the current state and roadmap for W3C standards for web applications on mobile, and, finally, by Alex Clark, Molecular Materials Informatics, Inc., who presented actual perspectives, known limitations, and some real examples of mobile applications for life sciences.

## Selection of best papers

Eleven papers were submitted for publication in this Supplement shortly after the conference. An Editorial Board was formed by paying attention that topics of submitted manuscripts were properly covered. It included the following Associated Editors:

• Davide Baù, Centre for Genomic Regulation (CRG), Barcelona, Spain

• Riccardo Bellazzi, University of Pavia, Italy

• Francisco Couto, University of Lisbon, Portugal

• Robert Davey, The Genome Analysis Centre, Norwich, United Kingdom

• Monika Heiner, Brandenburg University of Technology Cottbus, Germany

• Marco Masseroli, Politecnico di Milano, Italy

• Steve Pettifer, University of Manchester, United Kingdom

• Rafal Rak, University of Manchester, United Kingdom

• Andrea Splendiani, IntelliLeaf, United Kingdom

• Stefano Toppo, University of Padua, Italy

• Katy Wolstencroft, Leiden University, The Netherlands

Each Associate Editor managed the reviewing process for one paper, according to his/her expertise. At least two, but often three, referees were selected for each submission, and overall 27 referees were involved in the selection of papers. A two-step peer review procedure was adopted: some of the authors were invited to submit a revised version of their paper when it wasn't neither accepted nor rejected at the first step, according to the referees' comments and the associated editor recommendation. Associated Editors made a global assessment for papers assigned to each of them and provided the final recommendation for each paper. At the end of this process, six papers were accepted and they are now included in this Supplement.

## A short presentation of selected papers

Workshop topics included issues, methods, algorithms, and technologies for the design and development of tools and platforms able to provide semantic, social, and mobile applications supporting bioinformatics and the activities carried out in a biomedical laboratory. Not all topics were addressed by submissions and for this reason the six papers that were selected for this publication mainly relate to semantic tools.

In "Calculating semantic relatedness for biomedical use in a knowledge-poor environment" [10], Rybinski and Aldana-Montes present an original method for computing semantic relatedness between textual labels representing biological and medical concepts in a knowledge-poor context, i.e. without reference terminologies. The authors evaluate the effect of the parameters involved in the calculus for different benchmarks against state of the art results, showing that their method obtains results which are comparable, and often better, than state of the art methods.

Venco et al. developed a Laboratory Information Management System (LIMS) for Next-Generation Sequencing (NGS) data management and analysis needs of their laboratory, where they are sequencing about 2,000 samples per year submitted by ca. 150 different users. In their paper "SMITH: A LIMS for handling next-generation sequencing workflows" [11], they first present the need for flexibility and scalability, due to the frequent changes of their analysis protocols, that led them to the conception of SMITH (Sequencing Machine Information Tracking and Handling). A detailed presentation of the LIMS, both technical and operational, follows. A demo version of the system and the source code are available from the authors.

The work "LinkedISA: semantic representation of ISA-Tab experimental metadata" [12] is authored by the researchers that developed the Investigation/Study/Assay (ISA) metadata tracking framework, an open source system aimed at simplifying collection, curation, visualisation, storage and sharing of datasets according to existing standards. In this work, a novel methodology to transform data from the ISA-Tab format into the RDF format is introduced, as well as a new software component of the ISA framework, the linkedISA conversion tool, that relies on mappings from the ISA syntax to multiple community-defined, open ontologies. An evaluation of the resulting RDF representation is performed by running demonstration queries.

Soldatova et al. present "EXACT2: the semantics of biomedical protocols" [13]. In this paper, the authors introduce the EXACT2 (EXperimental ACTions) ontology aimed at capturing the semantics that is required for the reproducibility of biomedical protocols. The ontology was built after the manual inspection of hundreds biomedical protocols. Text mining tools have being used to translate protocols into a machine manageable format. The ability of EXACT2 to capture the semantics of biomedical procedures was evaluated in a use case. Finally, authors propose an EXACT2-based framework for translating protocols to a machine amenable format.

The paper "OntoGene web services for biomedical text mining" from Rinaldi et al. [14] reports on web services for biomedical text mining provided by the OntoGene project and on the OntoGene Document INspector (ODIN) annotation interface. Biocurators are increasingly interested in assistance of innovative computational tools for their curation needs, and such tools are becoming more and more reliable. The use of the BioCreative standard format for textual data interchange (BioC) is for sure a significant addition on this path. The use of web services is a very flexible way to include tools from multiple sources into practical annotation pipelines.

Möller et al. present "Community-driven development for computational biology at Sprints, Hackathons and Codefests" [15], a clear and incisive report on new ways for interactive meetings of software developers. Informal meetings have played a fundamental role for open source development, enabling interaction among developers of various levels of experience, as well as joint problem-solving work and hands-on training. These new forms promise to support the creation of new collaborations, and the spread of ideas and best practices not only for software development, but also for scientific research.

## List of abbreviations used

BioC: BioCreative standard for textual data interchange; ERCIM: European Research Consortium for Informatics and Mathematics; EXACT2: EXperimental ACTions; ICT: Information and Communication Technologies; ISA: Investigation/Study/Assay; LIMS: Laboratory Information Management System; NETTAB: Network Tools and Applications in Biology; ODIN: OntoGene Document Inspector; RDF: Resource Description Framework; SMITH: Sequencing Machine Information Tracking and Handling; W3C: World-Wide Web Consortium; XML: Extensible Markup Language.

## Competing interests

The authors declare that they have no competing interests.

## Authors' contributions

All authors discussed and agreed about the organization of the paper. PR wrote the paragraphs related to the NETTAB Workshops, while NC contributed to the description of the rationale of NETTAB 2013 and the related topic description. Each author wrote some of the presentations of papers. All authors read and agreed on the final version of the paper.

## Declarations

Publication of this article has been funded by the IRCCS AOU San Martino IST.

This article has been published as part of *BMC Bioinformatics *Volume 15 Supplement 14, 2014: Semantic, social, mobile applications for bioinformatics and biomedical laboratories: selected works from the 13th International Workshop on Network Tools and Applications in Biology (NETTAB 2013). The full contents of the supplement are available online at http://www.biomedcentral.com/bmcbioinformatics/supplements/15/S14.
